# Editorial: Surveying Antimicrobial Resistance: The New Complexity of the Problem

**DOI:** 10.3389/fmicb.2020.01144

**Published:** 2020-06-12

**Authors:** Gilberto Igrejas, José Luis Capelo, Carlos Lodeiro, Patrícia Poeta

**Affiliations:** ^1^Department of Genetics and Biotechnology, University of Trás-os-Montes and Alto Douro (UTAD), Vila Real, Portugal; ^2^Functional Genomics and Proteomics Unit, University of Trás-os-Montes and Alto Douro (UTAD), Vila Real, Portugal; ^3^Associate Laboratory for Green Chemistry (LAQV), Chemistry Department, Faculty of Science and Technology, University Nova of Lisbon, Costa da Caparica, Portugal; ^4^BIOSCOPE Group, LAQV@REQUIMTE, Chemistry Department, Faculty of Science and Technology, NOVA University of Lisbon, Almada, Portugal; ^5^Proteomass Scientific Society, Costa de Caparica, Portugal; ^6^Microbiology and Antibiotic Resistance Team (MicroART), Department of Veterinary Sciences, University of Trás-os-Montes and Alto Douro (UTAD), Vila Real, Portugal

**Keywords:** antibiotics, AMR, genomics, microbiology, omics

The 2nd International Caparica Conference in Antibiotic Resistance (IC2AR) was held in Caparica, Portugal from 11 to 15 June 2017. This very successful meeting had a clear One Health vision and attracted 216 attendees from 39 countries keen to exchange knowledge and expertise on diverse but interrelated topics. Formal contributions totaled over 131 oral presentations, 19 short presentations and 49 posters. The results and insights from this meeting are now being made accessible to the general scientific community in this special issue of the Frontiers in Microbiology Research Topic.

The introduction of penicillin in the 1940s, the start of the antibiotics era, has been recognized as one of the greatest advances in therapeutic medicine. However, according to the World Health Organization (WHO), antimicrobial resistant infections are now an increasing worldwide public health threat and a post-antibiotic era is imminent when even common infections and minor injuries could be fatal. Antimicrobial resistance (AMR) reduces the effectiveness of treatment and patients remain infected for a longer period, thereby increasing the potential to spread resistant microorganisms to others, according to WHO. Without effective antimicrobials to counter and prevent infections, other major achievements in modern medicine, such as organ transplantation, cancer chemotherapy and major surgery, risk being compromised. According to The State of the World's Antibiotics, two-thirds of the 100,000 tons of antibiotics produced globally each year are used in animal husbandry, and of the 27 antimicrobials used in animals, 18 are also used for human medicine. In terms of global sales in 2009, the top three antimicrobial classes for use in animals were macrolides, penicillins and tetracyclines, all of which are categorized as being critical for human medicine. The growth of global trade and travel allows resistant microorganisms to be spread rapidly to distant countries and continents, which threatens health security and risks damaging trade and economics.

AMR is becoming one of the most threatening public health issues worldwide. In Europe, the Mediterranean countries are most at risk, possibly due to a complex combination of antibiotic use practices, socio-economic factors and climate changes. For economies that rely heavily on tourism and export of food crops, the current situation is delicate. For the well-being and safety of the populations and for socio-economic stability, the increase in AMR must be reversed.

AMR infections in animals have negative outcomes on animal health, welfare, biosecurity and production. Growth promoting antimicrobials have been banned in the EU countries in 2006, however they are in widespread use in other countries outside the EU. Antibiotic use in animal production was highlighted as a risk factor in the development of antibiotic resistant bacteria that can be transferred to humans via several routes.

With the increasing resistance of bacterial pathogens to present-day antibiotics and the lack of a robust pipeline to generate novel antimicrobial substances, more innovative and efficient approaches are needed to develop anti-infective drugs. Proteomics and genomics technologies already offer sensitive and specific methods for identification of microbial food contaminants and their toxins. So, there is a lot to learn and discuss about these cutting-edge methods.

AMR within populations of different infectious agents is a worldwide public health threat. Already the available treatment options for common infections in some settings are becoming ineffective. There are now reports of bacterial resistance to all antibiotic classes used in either human or veterinary medicine, and in several cases, of an association between antibiotic use and the development of clinical resistance. To counter this emergent problem, the World Health Organization has appealed for urgent and concerted action by governments, health professionals, industry, civil society and patients to slow down the spread of drug resistance, limit its impact today, and so preserve medical advances for future generations.

The prevalence of AMR varies greatly between and within countries and between different pathogens. The widespread use of antimicrobial agents in human and veterinary medicine for therapeutic and prophylactic purposes has been identified as the main determinant for the emergence and spread of resistant bacteria. However, there are hardly any specific integrated studies that indicate how the risk could be limited. Progress has been made in recent years in understanding the AMR mechanisms underlying the emergence of the resistance genes and their spread, but there are still major gaps. Co-integrated research on resistance in animals and the environment together with in-depth pharmacokinetics and pharmacodynamics of antibiotics will contribute to this understanding. As One Health Initiatives get underway, a global perspective must be encouraged and maintained even for very focused investigations.

Livestock and the environment constitute AMR reservoirs and transmission routes to and from the human population. Environmental antibiotic resistance genes are spread then acquired by clinically relevant microorganisms. Many resistance genes are conveyed into pathogen genomes via mobile genetic elements such as plasmids, transposons or integrons, increasing the propagation of potentially resistant pathogens and the intricacies of these adaptive mechanisms are still the focus of investigation. This Research Topic presents original research on integrative and conjugative elements and the staphylococcal cassette chromosome, as well as new studies of resistance gene variants borne by plasmids or transposons, and characterization of the regulation of their gene expression.

Substantial progress has already been made in elucidating the basic regulatory networks that endow bacteria with their extraordinary capacity to adapt to a diversity of lifestyles and external stress factors. The articles collated here describe microbial life in a vast spectrum of natural and manmade settings. Just to illustrate this variety, micro-organism samples studied have been collected from 2 m depth of sediment on the Red Sea coast and from the International Space Station orbiting 400 km above the Earth's surface (Rehman and Leiknes; Sobisch et al.). Microbes from aquatic ecosystems of seas, rivers and wetlands have also been analyzed (Rehman and Leiknes; Tuo et al.; Sen et al.). Farming and food production contexts cover organic, conventional and intensive agriculture (Zheng et al.; Cadena et al.; Liu et al.; Miranda et al.; Armalyte et al.; McMillan et al.; Zajac et al.). The non-food animal hosts studied range from wild primates in Brazilian forests and flocks of crows over US farmland to pet cats and dogs in Spanish homes (Grassotti et al.; Roberts et al.; Gómez-Sanz et al.; Sen et al.). Clinical research comes from hospitals in a range of different healthcare systems with presentation of a range of pathologies and includes the analysis of historical specimens providing some longer-term perspective that is valuable in depicting the timescale of mutation and spread of resistance (Manageiro et al.; Bostanghadiri et al.; Ferreira et al.; Palmeiro et al.; Pinto et al.). The fundamental ecology of microbiota is still a strong focus with investigations of quorum sensing, biofilm formation, stress responses and resistance mechanisms (Knight et al.; Rehman and Leiknes; Martins et al.; Sobisch et al.; Yang et al.). Without a robust pipeline to generate novel antimicrobial substances, more innovative and efficient approaches are needed to develop anti-infective drugs and some of these will be based on specific biological functions or the dynamics and interactions of microbial populations (Grassotti et al.; Igrejas et al.; Jäger et al.; Troiano et al.; Zhang et al.; Armalyte et al.).

Improvement of food safety standards helps to strengthen the competitiveness of the food industry. To achieve this, microbial food contamination, risks and exposures must be analyzed, assessed, monitored, controlled and traced throughout the food supply chains from production and storage to processing, packaging, distribution, catering, and preparation at home. Many of the papers published here deal with some stage or aspect of this complex process (Dominguez et al.; Cui et al.; McMillan et al.; Zajac et al.). It is important to design research that contributes to ensuring the safety of food of animal origin while addressing the sustainability of food production, supply and consumption, along the whole food chain and related services from field to fork. When dealing with the issue of safe food, healthy diets and sustainable consumption, the control of foodborne outbreaks must always be a priority (Isidro et al.). Current research focuses strongly on the detection of foodborne pathogens and specific spoilage organisms from food of animal origin along different production chains (slaughterhouses, restaurants, meat product manufacturers, fisheries). Important microbiological hazards responsible for foodborne outbreaks are analyzed, such as those involving *Salmonella* sp., *Campylobacter* spp., *E. coli, Listeria* spp., or *Aeromonas* spp. (Bai et al.; Hormeño et al.; Peng et al.; Beshiru et al.; Cyoia et al.; Islam et al.). Researchers will continue to develop new approaches to analyze and interpret more complex and emerging microbial pathogens using molecular, serotyping and phylogenetic methods. Expected developments will be in pinpointing and surveying prevalence, contamination sources, public health risks, and strategies to improve food safety and quality (Dandachi et al.; Igrejas et al.; Domokos et al.; Zeineldin et al.). For example, packaging, temperature treatments, and traditional methods for meat preservation (fermentation, drying, spices and herbs, wine) may be revisited with modern technologies (Sparo et al.; Igrejas et al.). With the policies to reduce the use of additives and promote environmentally sustainable production of meat products, research to develop and validate organic preservation procedures will be necessary (Beshiru et al.; Li et al.; Sen et al.; Wieczorek et al.).

On the subject of food safety, studies on the resurgence of AMR as a pandemic threat must be included. Presently and in the near future, antimicrobial peptides produced by different microorganisms will be characterized to generate novel applications in human and veterinary medicine and in food conservation. Such discoveries will also facilitate research on antibiotic resistance and molecular characterization of virulence factors in microbiota from different ecological niches. Antimicrobial peptides are indeed the subjects of original research, review, and opinion articles published here which give some indication of current strategic thinking (Pizzolato-Cezar et al.; Vasilchenko and Rogozhin).

Proteomics and genomics technologies already offer sensitive and specific methods for identification of microbial food contaminants and their toxins. A perusal of the techniques and technologies used in AMR research shows that whole-genome sequencing is now well-entrenched alongside conventional molecular and microbiological techniques, an approach that is clearly increasing the diversity, depth and pace of AMR monitoring and basic research. Impact studies that analyze and assess some of the cumulated economic, epidemiological or environmental data are also featured here (Annavajhala et al.).

To summarize, this Research Topic brings together a group of leading researchers from all over the world who have described different aspects of AMR patterns found in diverse ecosystems. The articles address the epidemiology of resistance in animal and zoonotic pathogens, mobile elements containing resistance genes, the omics of AMR, emerging AMR mechanisms, control of resistant infections, establishing antimicrobial use and resistance surveillance systems, and alternative strategies to overcome the problem of AMR worldwide. In this conference an attempt was made to present the latest research on possibilities to manage this question. The meeting carried out an integrated approach to research and presented a universal vision of the importance of antimicrobial resistance in different ecosystems and what can be done about it.

We want to thank the reviewers for their many thoughtful and insightful comments, and the authors for their high-quality contributions. In closing, we would like to encourage readers to participate in the 4th edition of the International Caparica Conference in Antibiotic Resistance to be held in 2021 (http://www.bioscopegroup.org/index.php/congresses).

## Author Contributions

All authors listed have made a substantial, direct and intellectual contribution to the work, and approved it for publication.

## Conflict of Interest

The authors declare that the research was conducted in the absence of any commercial or financial relationships that could be construed as a potential conflict of interest.

